# Asaprol

**Published:** 1892-08-27

**Authors:** 


### THERAPEUTICS.

### ASAPROL.

At the laboratory of the Hôpital Cochin, Dr. Stackler has made some observations on a derivative of α mono-sulphate of β naphthol, C. 10 H. 6 $<\begin{matrix}\alpha\ SO.\ 3\\ \beta\ OH.\end{matrix}$ in the form of a lime salt. It was manufactured having regard to the doctor's experiences with existing antiseptics by M. Bang, by whom two necessary conditions were considered essential, viz., only the absolutely pure β naphthol should be utilised in its preparation, and that the α and β forms of the salt should be completely isolated.

This substance has been investigated under the name of asaprol (α not σαπρὸς—putrescence) in the laboratory and in the clinic of M. Dujardin-Beaumetz. It is neutral in reaction, very soluble in water and alcohol, unaltered by heat, non-irritating, tolerated by the intestinal tract, hardly at all toxic, and is rapidly eliminated. Injected hypodermically in guinea-pigs it produced no local trouble.

Dr. Stackler, with Dr. Dujardin-Beaumetz, investigated the action of the substance upon the various microbal culture preparations, and found that in five c.c. of bouillon the culture is hindered, with ten c.c. of asaprol in the case of asiatic cholera, herpes tonsurans, typhoid fever bacillus, it is stopped; with 15 cent. in asiatic cholera, herpes tonsurans, typhoid fever bacillus, streptococcus aureus, charbon, and the pyocyaneus bacillus is hindered in growth. With 30 c.c. it is stopped for all these microbes. In the case of man, this product, ingested in the dose of one to four grammes, is a medicament used with advantage in the various forms of rheumatism; it does not diminish the amount of urinary secretion, not infrequently it actually increases it; it is antipyretic in various febrile states in typhoid fever; in acute poly-articular rheumatism it has produced a rapid cure.*

* Bull. gén. de Ther. Mar. 13, 1892.

